# Rethinking the causes of pilonidal sinus disease: a matched cohort study

**DOI:** 10.1038/s41598-021-85830-1

**Published:** 2021-03-18

**Authors:** Dietrich Doll, Imke Brengelmann, Patrick Schober, Andreas Ommer, Friederike Bosche, Apostolos E. Papalois, Sven Petersen, Dirk Wilhelm, Johannes Jongen, Markus M. Luedi

**Affiliations:** 1 Department of Procto-Surgery and Pilonidal Sinus Research Group, Germany, St Marienhospital Vechta, Academic Teaching Hospital of the Medical School Hannover, Vechta, Germany; 2 Department of Anesthesiology, Amsterdam University Medical Centers, Vrije Universiteit Amsterdam, Amsterdam, The Netherlands; 3 End- und Dickdarm-Zentrum Essen, Essen, Germany; 4 ELPEN Pharmaceutical Research and Experimental Center, Athens, Greece; 5 Department of General, Visceral and Vascular Surgery, Asklepios Klinik Altona, Hamburg, Germany; 6 Department of Surgery, Klinikum Rechts der Isar, Munich, Germany; 7 Private Practice, Kiel, Germany; 8 Department of Anaesthesiology and Pain Medicine, Inselspital, Bern University Hospital, University of Bern, Bern, Switzerland

**Keywords:** Medical research, Risk factors, Signs and symptoms

## Abstract

Our understanding of pilonidal sinus disease (PSD) is based on a paper published 29 years ago by Karydakis. Since then, surgeons have been taught that hair more easily penetrates wet skin, leading to the assumption that sweating promotes PSD. This postulate, however, has never been proven. Thus we used pilocarpine iontophoresis to assess sweating in the glabella sacralis. 100 patients treated for PSD and 100 controls were matched for sex, age and body mass index (BMI). Pilocarpine iontophoresis was performed for 5 min, followed by 15 min of sweat collection. PSD patients sweated less than their matched pairs (18.4 ± 1.6 µl vs. 24.2 ± 2.1 µl, *p* = 0.03). Men sweated more than women (22.2 ± 1.2 µl vs. 15.0 ± 1.0 µl in non-PSD patients (*p* < 0.0001) and 20.0 ± 1.9 µl vs. 11.9 ± 2.0 µl in PSD patients (*p* = 0.051)). And regular exercisers sweated more than non-exercisers (29.1 ± 2.9 µl vs. 18.5 ± 1.6 µl, *p* = 0.0006 for men and 20.7 ± 2.3 µl vs. 11.4 ± 1.4 µl, *p* = 0.0005 for women). PSD patients sweat less than matched controls. Thus sweating may have a protective effect in PSD rather than being a risk factor.

## Introduction

Pilonidal sinus disease (PSD) is a surgical-dermatological condition which is associated with a number of misconceptions. It is an acute or chronic infection of the subcutis responsible for an ever-increasing share of operations, mostly in young males. Important factors for the pathogenesis are the presence of a skin fold as well as straight, stiff, sharp, cut hair that penetrates the upper intergluteal fold (IGF)^1–3^. However, the pathomechanism of PSD has never been fully clarified.

In 1992, Karydakis postulated that a soft and macerated skin surface would be more prone to injury and to insertion of sharp hair than a dry and impermeable one^4^. Surgeons have been taught Karydakis’ view of the factors that promote PSD, including 5 hair factors (mechanical characteristics of the hair), 3 force factors (factors promoting the force of insertion of the hair) and 7 vulnerability factors^4^, seen in Table 1.

**Table 1:** Secondary factors in pilonidal sinus disease as defined by Karydakis in 1992^4^ (reproduced with permission from John Wiley and Sons/ANZ J Surg, License No 4647550740255, obtained 14.8.2019).

H factors	H1	The number of loose hairs collected in the natal cleft
	H2	The more or less acuteness of the root end of hair
	H3	The kind of hair (tough or silky)
	H4	The shape of the hair (straight hair, not curly, is the type liable to insert)
	H5	Scaliness of hair—more marked at age 10–22 years
F factors	F1	Depth
	F2	Narrowness of the natal cleft
	F3	Friction with movements between the sides of the cleft
V factors	V1	Softness
	V2	Maceration
	V3	Erosion
	V4	Splints
	V5	Wide pores
	V6	Wounds
	V7	Scars at the natal cleft

In the past nineteen decades, allegedly insufficient hygiene^5^, sweating as such, and sweating due to obesity have been blamed for more frequent PSD—teaching points which are routinely covered in classic educational manuscripts globally. While poor hygiene has been disproven as a cause in the military population^6^ and in the civilian population^5^, obesity and sweating remain assumed risk factors, and globally it is taught that they cause PSD.

We investigated the sweating ability of PSD patients and their matched pair counterparts. Our study hypothesis H_α_ was that pilonidal patients sweat significantly more in the lumbar region above the natal cleft if stimulated in a standardised fashion with pilocarpine iontophoresis.

## Results

**Figure 1:**
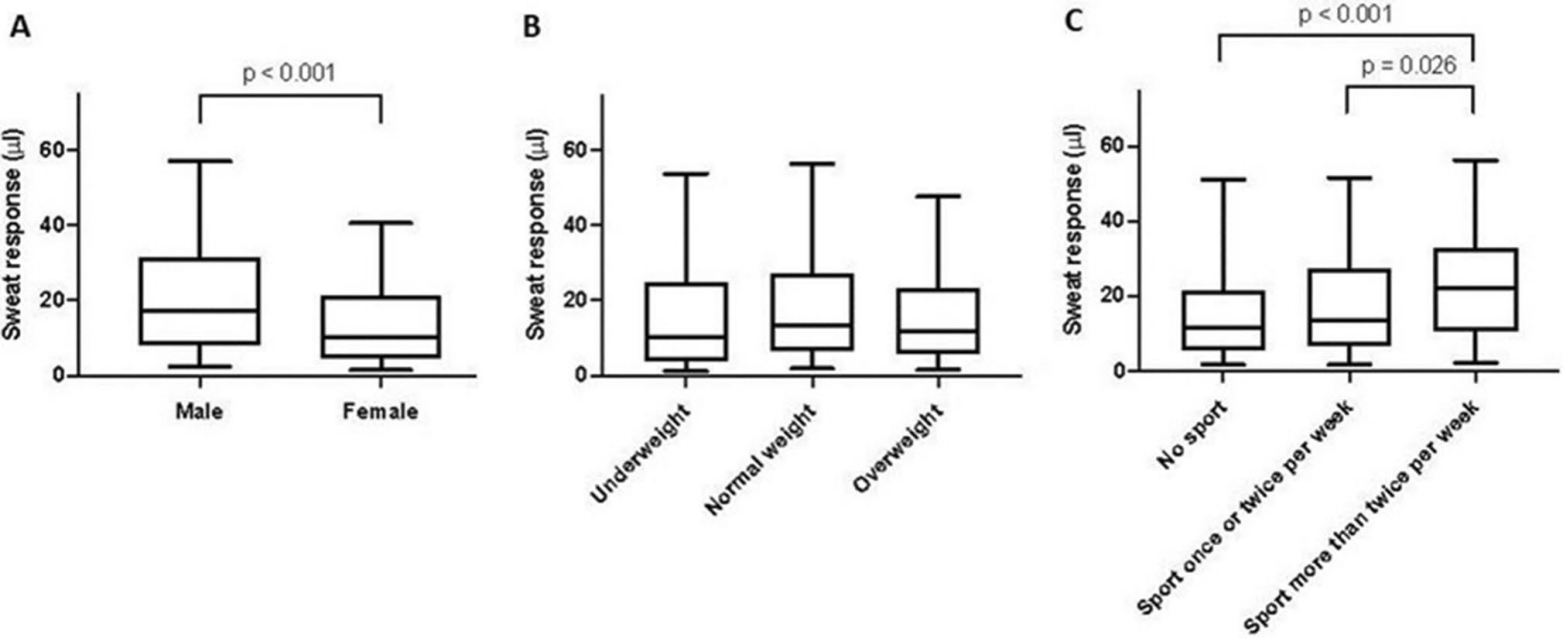
Sweat response in an unadjusted comparison. Box-and-Whisker plots showing the unadjusted difference in sweat response between male and female subjects (panel 1**A**), across different BMI categories (panel 1** B**) and across exercise categories (panel 1**C**). The boxes show the medians and quartiles, while the whiskers extend from the 5th to the 95th percentile. *p* values are based on Mann–Whitney U tests, with Bonferroni correction for post-hoc comparisons in Panel C.

459 patients (232 females (41.5%) and 327 males (58.5%)) were available for analysis (mean age of 36.8 ± 18.8 years, BMI of 25.6 ± 5.4 kg/m^2^). PSD patients and controls did not differ in sweat response in an unadjusted comparison (*p* = 0.816). However, males sweated significantly more than females (*p* <0.001, Fig. 1A). Additionally, older age was associated with a lower sweat response (*p* <0.001). BMI was only analysed in > 15-year-olds because it is co-linear with age at childhood and was not associated with the sweat response overall (*p* <0.236, Fig. 1B). Analysis of weekly exercise suggested an increase in sweating capability with increased physical activity overall (*p* <0.001, Fig. 1C). In particular, subjects who exercised more than twice a week had a greater sweat response than non-exercising individuals, measured with pilocarpine iontophoresis tests (Bonferroni adjusted post-hoc *p* <0.001) and compared to subjects who exercised less (Bonferroni adjusted post-hoc *p* = 0.026). While individuals who exercised once or twice per week had a higher sweat response in our sample than patients who did not exercise at all, this difference was not significant (Bonferroni adjusted post-hoc *p* = 0.455).

### Adjusted analyses of the primary outcome

**Figure 2:**
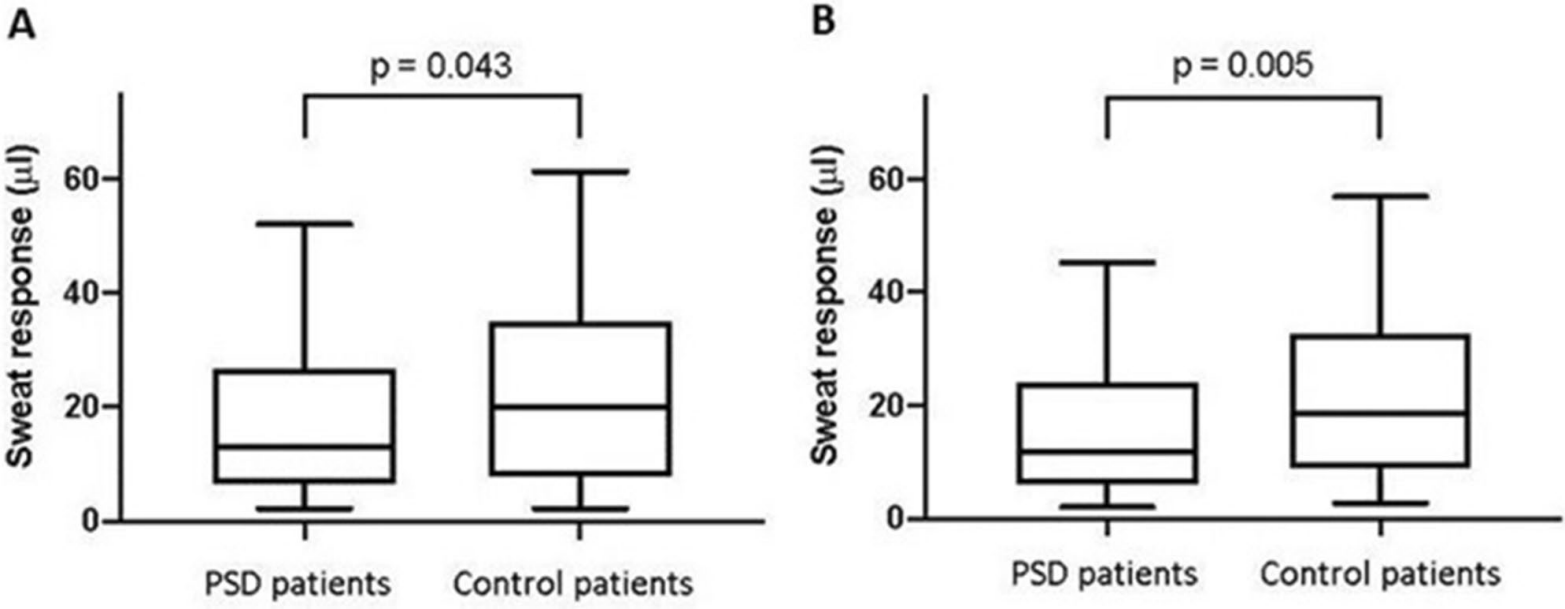
Sweat response in a manually and propensity score matched comparison. Box-and-Whisker plots of the primary outcome (sweat response) in the manually matched (panel 2**A**) and propensity score matched (panel 2**B**) cohorts. The boxes show the medians and quartiles, while the whiskers extend from the 5th to the 95th percentile. *p* values are based on the Wilcoxon signed-rank test.

One hundred PSD patients (81 males and 19 females) took part in the study and were manually matched with 100 controls of healthy volunteers of all ages. The groups were well balanced with respect to gender, age and BMI (Table 2). Sweat response in PSD patients (13.2 µl [6.5–26.7]) was significantly lower than in controls (20.0 µl [8.1–35.0], *p* = 0.043, Fig. 2A).

**Table 2:** Distribution of baseline characteristics before and after matching.

Full patient cohort	PSD patients (n = 100)	Healthy patients (n = 459)	Absolute MSD
Gender (N (%))			0.611
Male	81 (81.0%)	246 (53.6%)	
Female	19 (19.0%)	213 (46.4%)	
Age (years)	27.8 ± 10.5	38.8 ± 19.6	0.699
BMI (kg/m^2^)	26.9 ± 4.4	25.4 ± 5.5	0.312
Exercise (N (%))			
No exercise	45 (45.0%)	146 (31.8%)	0.363
Exercise once or twice a week	20 (20.0%)	124 (27.0%)	
Exercise more than twice a week	25 (25.0%)	101 (22.0%)	
Missing	10 (10.0%)	88 (19.2%)	

Manually matched samples	PSD group (n = 100)	Matched control group (n = 100)	Absolute MSD
Gender (N (%))			0.000
Male	81 (81.0%)	81 (81.0%)	
Female	19 (19.0%)	19 (19.0%)	
Age (years)	27.8 ± 10.5	27.9 ± 10.2	0.009
BMI (kg/m^2^)	26.9 ± 4.4	26.5 ± 4.3	0.093
Exercise (N (%))			0.416
No exercise	45 (45.0%)	35 (35.0%)	
Exercise once or twice a week	20 (20.0%)	23 (23.0%)	
Exercise more than twice a week	25 (25.0%)	39 (39.0%)	
Missing	10 (10.0%)	3 (3.0%)	

Propensity-score matched sample	PSD group (n = 90)	Matched control group (n = 90)	Absolute MSD
Gender (N (%))			0.053
Male	71 (78.9%)	69 (76.7%)	
Female	19 (21.1%)	21 (23.3%)	
Age (years)	27.5 ± 10.5	27.5 ± 11.*7*	0.000
BMI (kg/m^2^)	27.1 ± 4.5	27.2 ± 6.2	0.024
Exercise (N (%))			0.100
No exercise	45 (50%)	43 (47.8%)	
Exercise once or twice a week	20 (22.2%)	18 (20.0%)	
Exercise more than twice a week	25 (27.8%)	29 (32.2%)	
Missing	0	0

The propensity score matched sample that additionally adjusted for the level of exercise was also well balanced (Table 2) and provided consistent evidence of a lower sweat response in PSD patients (11.9 µl [6.0–24.0]) than matched controls (18.6 µl [8.9–32.3], *p* = 0.005, Fig. 2B). Likewise, the linear regression model including all 459 patients with complete data on all of the potentially confounding covariates (age, sex, BMI and exercise) showed a lower log-sweat response in PSD patients compared to controls (*p* = 0.003, Table 3). All three techniques that adjust for confounding consistently demonstrated less sweating in PSD patients.

**Table 3:** Linear regression of the natural logarithm of sweat response on PSD, gender, age, BMI and exercise.

	Coefficient	95% Confidence interval	*p* Value
Pilonidal sinus disease			
Yes	− 0.345	− 0.575, − 0.115	0.003
Gender			
Male	0.556	0.374, 0.736	< 0.001
Age (years)	− 0.007	− 0.012, − 0.002	0.008
BMI (kg/m^2^)	− 0.035	− 0.053, − 0.017	< 0.001
Exercise		0.151	
No exercise	(reference)		
Exercise once or twice a week	0.126	− 0.083, 0.335	0.238
Exercise more than twice a week	0.221	− 0.006, 0.447	0.056
Intercept	3.34		

BMI = body mass index.

## Discussion

While a succession of review articles have ranked “landmark” articles in the fields of general surgery, clinical dermatology and surgical education^7,8^, Karydakis’ article on PSD^4^ has never been challenged. For decades surgeons have assumed that maceration of the IGF is a risk factor for PSD and that pilonidal patients sweat more than people without the disease. Our analysis adjusting for confounding disproves this. We proved that sweat production in the lumbar area is significantly lower in PSD patients than in controls, leading us to reject our alternative hypothesis. Thus our findings overturn long-standing assumptions^4^ about the genesis of PSD. The insertion of hair takes place during a “vulnerable time window” around puberty^9,10^. PSD becomes symptomatic in combination with sweating and repetitive mechanical stress^11,12^. Sharp hair fragments reaching the lumbar area shortly after hair cutting may be a factor contributing to PSD^13^, especially in the military setting, where short hair is typical^12,14–16^.

What we did find is that sweat response is lower in females than in males, even after adjusting for BMI and age (Table 3). While we found increased sweating capacity in people who exercise more in the unadjusted analysis, the effect disappears in the regression model adjusted for age and BMI.

Although sweat does not harm the skin per se, it can promote a moist environment and enable adhesion of short cut hairs in the IGF, leading to longer contact times^17^ and higher injection rates^1,18^, thereby coinciding with higher incidence of PSD. If the IGF were totally dry, no hair would be retained by adhesion. Copious sweating would tend to wash the loose hair fragments downwards, keeping exposure time short. However, we did not see any of these effects in our German PSD cohort. PSD sweat response did not exceed the sweating in the matched pairs.

Males do sweat more, but as their body muscle mass is one third greater than that of their female counterparts, their ability to produce heat is greater, and their cooling system is adequately adapted. Sweating can be triggered by physical exercise or regular exposure to hot environments^19^, but exercise did not make any difference between PSD patients and their age-matched group in our study, following adjustment for confounding. Three different statistical techniques that adjust for confounding consistently demonstrated less sweating in PSD patients.

Habits leading to sweating tend to differ from country to country. For example, application of moisture on a daily basis is common in the Scandinavian countries, where excessive sweating in a sauna is quite popular. The incidence of PSD there is 26/100,000^20^. Thus sauna use and a Scandinavian lifestyle do not expose people to a higher incidence of PSD than for example in Turkey, where saunas are scarce^12^. Moist skin is also quite common in the tropical belt around the equator, a geographical area with an extremely low risk of PSD. Likewise, swimmers are not known to be prone to PSD, and regular sauna use^21^, bathing^22^ and showering^23^ may have protective effects.

Axial strength of the hair is reduced by 15–20% within 5 min when hair is wet^1^, while force is recovered after 30 min of drying. The highest force values and the most likely time of injection occur shortly after a dry haircut, when the sharp, dry, occipital hair fragments^18,24^ come in contact with the IGF^13^.

Visible skin changes occur following prolonged contact with moisture. It is unclear whether this weakens the skin, or if resistance is diminished. If skin´s perforation resistance stays the same while the skin is wet, and axial hair force is diminished, then moisture–either sweat or water–would have a protective effect in terms of PSD.

The Macroduct Sweat Collection Test was chosen because it is known to reliably evaluate sweat production of the skin^25,26^. Pilocarpine-induced sweat tests stimulate only muscarinically transmitted sweating, whereas central sweating requires nicotinic synaptic transmission. Central sweating is stimulated by thermoregulatory centres. Exercise leads to elevation of body temperature, triggering centrally induced production of sweat. This latter method is perfect for measurements in athletes, but unfortunately excludes several groups from participation in a study, including the young and the elderly, as well as patients with cardiocirculatory limitations, patients with hip and knee disease, and patients who have had surgery recently^27^. Induction of pilocarpine-mediated sweating does not cause significant cardiovascular stress, does not require physical exertion, and has been applied in newborns and frail subjects^28^.

Our findings are limited by the fact that only a northern German cohort was studied. As mentioned previously, sweating may be different in other populations. However, the matched controls in our study provide a reliable picture of this cohort. Further, we did not standardise the participants’ hydration, which may have influenced their sweating. Yet it is unlikely that the controls have a different hydration status, because PSD is a locally limited disease without systemic impact. Finally, we only measured the ability to sweat based on pilocarpine induction, which might not perfectly correlate with the effective sweat production observed under regular circumstances and in daily life.

## Conclusion

In conclusion, our findings present a different picture of pilonidal disease than the one provided by Karydakis. Pilonidal patients don’t sweat more than their gender-, age-, BMI- and exercise-matched controls, and the alternative hypothesis H_α_ that pilonidal patients sweat significantly more in the lumbar region should be rejected.

Our data highlights the importance of educators evaluating the materials they provide and procedures they teach. Future research into PSD should focus on skin texture changes, hormonal changes during puberty, age-related sweating, hair strength, and skin-to-hair-strength ratio changes.

### Patients and methods

The ethics committee of the medical association of Niedersachsen, Berliner Allee 20, 30,175 Hannover, Germany (Prof. Dr. med. Andreas Creutzig, chair) fully and unanimously approved the study based on § 15 of the Niedersachsen Medical Association’s professional code of conduct.

Consenting patients treated for PSD and controls at the St. Marienhospital in Vechta, Germany, were studied. In this matched cohort study, 100 PSD patients were compared to 100 healthy non-PSD subjects aged 5–79 years belonging to a northern German population cohort (n = 459 persons; 246 males/213 females). Patients with thyroid disease, pain greater than VAS 3, and diseases with known or suspected temperature or fluid imbalance (fever, kidney disease, Ileus, dehydration, endocrine disorders) were ineligible for the study.

The pilonidal patients were matched with their healthy non-PSD controls for sex, age at time of testing, and body mass index (BMI) in the order described. The quality of matching was assured by calculating absolute Standardised Mean Differences (SMD).

Sweat testing was performed using the Macroduct Sweat Collection System (Webster Sweat Inducer, electrodes for iontophoresis, SS-032G pilocarpine gel discs and sweat-collecting device, Kreienbaum Neoscience GmbH, Langenfeld, Germany), following a standardised sweat collection procedure and constant room temperature. The test area was restricted to the lumbar region, with the red electrode positioned midway between the fossae sacrales (Supplemental Fig. S1). Cleaning of this area with alcohol swabs was avoided to exclude unnecessary skin perfusion changes. Truncal sweat production was induced by pilocarpine iontophoresis on the glabella sacralis (GS, red electrode) and a finger breadth above (black electrode). Iontophoretic current was fixed at 1.5 mA for 5 min using a Webster sweat inducer (model 3700). Pilocarpine was delivered by reagent-impregnated (0.5% pilocarpine) solid agar gel disks (Pilogel disks). This procedure has been shown to elicit sweat production in a reliable and standardised manner^29–31^. Sweat was harvested for 15 min after standardised stimulation with a Macroduct sweat collector and measured by determining the length of the fixed diameter plastic tube filled with sweat using the standardised scale. Immediately after removal of the sweat collector system, the remaining small amounts of fluid present on the skin (at the area where the sweat collector contacted the skin) were collected with a dry swab pre-weighed on an air movement shielded Sartorius scale (model 1201 MP2, Sartorius). The weight of the swab plus the fluid was added to the fluid collected and documented (Excel, 2003, Microsoft Corp., Richmond, Virginia, USA).

Terminal hair was judged to be present or absent in the PSD patients and their matched pairs within three defined body regions, as hair at the occipital scalp overlying the *protuberantia occipitalis externa* (POE), in the lumbar region (*GS*) and in the upper third of IGF. Vellus hair (lanugo; soft curly white hair shorter than 2 mm) was not assessed; regions with solely or no Vellus hair were defined “Terminal bold”. All subjects were classified according to the World Health Organization (WHO) body mass index (BMI) category 1 < 18.5 kg/m^2^, category 2 18.5 kg/m^2^ to < 25 kg/m^2^, and category 3 > 25 kg/m^2^^32^.

For category 3 we combined the BMIs of WHO pre-obese patients and those with class I-III obesity. We did not use waist circumference measurements, which are known to be a good estimator of intraabdominal fat tissue and cardiovascular risk^33^, but are less accurate for body weight-induced exertion, as they do not take height and related weight into account^34^.

### Statistical methods

To test our hypothesis that pilonidal patients sweat significantly more in the lumbar region, the distribution of the data was assessed with histograms, Q-Q plots and Shapiro–Wilk tests. Continuous data are reported as mean ± standard deviation or median [quartiles] as appropriate, and categorical data are reported as counts and percentages.

#### Exploratory unadjusted analyses

The unadjusted difference in the sweat response between all patients with and without PSD and gender differences were evaluated with Mann–Whitney U tests^35^. The relationship between age and the natural logarithm of the sweat response was assessed in a linear regression model, in which age was modelled using restricted cubic splines to account for a possible non-linear relationship. A likelihood ratio test was used to test for the overall effect of age. The score was logarithmised to achieve approximately normally distributed residuals in the regression model. Differences across BMI categories or across exercise levels were compared with Kruskal–Wallis tests. Pairwise post-hoc comparisons were performed with Mann–Whitney U tests, and *p* values of these analyses were adjusted for multiple comparisons with the Bonferroni technique.

#### Adjusted analyses of the primary outcome

To adjust for confounding factors that could be related to both PSD and sweating, the 100 PSD patients in the dataset were manually matched to 100 similar controls based on age, sex, and BMI, as described above. The adequacy of the matching in balancing the potential confounding factors between the groups was determined by calculating absolute Standardised Mean Differences (SMD). At a sample size of 100 per group, an absolute SMD of larger than 0.28 would indicate imbalance^36^. The sweat-response score was compared between the matched patients^37^ using a Wilcoxon matched-pairs signed-rank test^37^.

To test the validity of our manual matching approach in controlling for confounding and to control for exercise as a potential confounder, we additionally used propensity score matching and linear regression as sensitivity analyses^38^. Propensity scores were estimated with logistic regression, using age, sex, BMI and exercise as independent variables. Patients were matched 1:1 on the logit of the propensity score with a calliper width of 0.2 standard deviations of the logit and without replacement^36^. A total of 100 PDS patients were matched to 100 controls. The sweat-response score was compared between the matched patients as described above for the matched pairs. Using linear regression, the natural logarithm of the sweat-response score was compared between patients with and without PSD across the full sample while adjusting for age, sex, BMI and exercise^39^.

Statistical analyses were performed in Stata/IC 16.0 (StataCorp, College Station, TX, USA). A sample size of 100 per group in the matched-pairs analysis is sufficient to detect a moderate effect size (Cohen’s d = 0.3) with 80% power at a significance level of 0.05. Two-sided *p* values < 0.05 were considered statistically significant.

### Ethics

All procedures performed in studies involving human participants were in accordance with the ethical standards of the institutional and/or national research committee and with the 1964 Helsinki declaration and its later amendments or comparable ethical standards. This study did not contain any interventions that could cause harm to human participants. Nevertheless, informed consent was obtained from all patients prior to the sweat tests. Ethical approval of the study was obtained in advance through formal application to and approval by the IRB of the Ärztekammer Hannover (BO/33/2016 from 8.12.2016). The IRB contact is Prof. Dr. med. Andreas Creutzig, Chair of the ethics committee of the Medical Association of Niedersachsen, Berliner Allee 20, 30175 Hannover, Germany, Phone: +49 511 380 22 08, Fax: +49 511 380 21 19, Email: ethikkommission@aekn.de.

## Supplementary Information

Supplementary Figure

## References

[1] Doll D (2017). Strength of occipital hair as an explanation for pilonidal sinus disease caused by intruding hair. DCR.

[2] Gosselink, M. & Ctercteko, G. The role of hair in the pathogenesis of pilonidal disease. *ESCP Teach. Pilonidal Sinus* **12** (2017). https://www.escp.eu.com/news/focus-on/pilonidal-disease/1550-the-role-of-hair-in-the-pathogenesis-of-pilonidal-disease-martijn-gosselink-and-grahame-ctercteko.

[3] Page BH (1969). The entry of hair into a pilonidal sinus. Br. J. Surg..

[4] Karydakis GE (1992). Easy and successful treatment of pilonidal sinus after explanation of its causative process. Aust. N. Z. J. Surg..

[5] Doll D (2015). Stop insulting the patient: neither incidence nor recurrence in pilonidal sinus disease is linked to personal hygiene. PSJ.

[6] Favre R, Delacroix P (1964). Apropos of 1,110 cases of pilonidal disease of Coccy-Perineal localization. Mem. Acad. Chir. (Paris).

[7] Wohlauer MV (2013). Review of influential articles in surgical education: 2002–2012. J. Grad. Med. Educ..

[8] Derossis AM, DaRosa DA, Dutta S, Dunnington GL (2000). A ten-year analysis of surgical education research. Am. J. Surg..

[9] Doll D (2008). Time and rate of sinus formation in pilonidal sinus disease. Int. J. Colorectal Dis..

[10] Ardelt M (2017). Puberty is a major factor in pilonidal sinus disease: Gender-specific investigations of case number development in Germany from 2007 until 2015. Chirurg.

[11] Patey D (1971). Pilonidal sinus–or 'jeep disease'. Nurs. Times.

[12] Akinci OF, Bozer M, Uzunköy A, Düzgün SA, Coskun A (1999). Incidence and aetiological factors in pilonidal sinus among Turkish soldiers. Eur. J. Surg..

[13] Doll D (2019). Immediate cut hair translocation to the intergluteal fold in the hairdressers shop – another link to pilonidal sinus disease. PSJ.

[14] Fabre A (1961). Technic for simple an radical treatment of sacrococcygeal fistulas and cysts. La Presse médicale.

[15] Evers T (2011). Trends in incidence and long-term recurrence rate of pilonidal sinus disease and analysis of associated influencing factors. Zhonghua Wai Ke Za Zhi.

[16] Karydakis G (1973). The problem of pilonidal cyst in the Greek Armed Forces (Greek). Hell. Arm. Forces Med. Rev..

[17] Akinci OF (2009). Natal cleft deeper in patients with pilonidal sinus: implications for choice of surgical procedure. DCR.

[18] Bosche F (2018). The hair in the sinus: sharp-ended rootless head hair fragments can be found in large amounts in pilonidal sinus nests. World J. Surg..

[19] Wells CL, Constable SH, Haan AL (1980). Training and acclimatization: effects on responses to exercise in a desert environment. Aviat. Space Environ. Med..

[20] Sondenaa K, Andersen E, Nesvik I, Soreide JA (1995). Patient characteristics and symptoms in chronic pilonidal sinus disease. Int. J. Colorectal Dis..

[21] Kowatzki D (2008). Effect of regular sauna on epidermal barrier function and stratum corneum water-holding capacity in vivo in humans: a controlled study. Dermatology.

[22] Bolandparvaz S (2012). Evaluation of the risk factors of pilonidal sinus: a single center experience. Turk. J. Gastroenterol..

[23] Harlak A (2010). Sacrococcygeal pilonidal disease: analysis of previously proposed risk factors. Clinics (Sao Paulo).

[24] Doll D (2018). The presence of occipital hair in the pilonidal sinus cavity-a triple approach to proof. Int. J. Colorectal Dis..

[25] Aqil B (2014). Implementation of a quality improvement program to improve sweat test performance in a pediatric hospital. Arch. Pathol. Lab. Med..

[26] Mastella G, Di Cesare G, Borruso A, Menin L, Zanolla L (2000). Reliability of sweat-testing by the Macroduct collection method combined with conductivity analysis in comparison with the classic Gibson and Cooke technique. Acta. Paediatr..

[27] Hjortskov N, Jepsen LT, Nielsen B, Juul A, Skakkebaek NE (1995). Pilocarpine iontophoresis test: an index of physiological sweat secretion?. Clin. Physiol..

[28] Vernooij-vanLangen A (2015). Clinical evaluation of the Nanoduct sweat test system in the diagnosis of cystic fibrosis after newborn screening. Eur. J. Pediatr..

[29] Shwachman H, Mahmoodian A (1967). Pilocarpine iontophoresis sweat testing results of seven years' experience. Bibl. Paediatr..

[30] Warren RH, Jones B, Heffington R (1983). Diagnostic testing in cystic fibrosis. J. Ark. Med. Soc..

[31] Barnes GL, Vaelioja L, McShane S (1988). Sweat testing by capillary collection and osmometry: suitability of the Wescor Macroduct System for screening suspected cystic fibrosis patients. Aust. Paediatr. J..

[32] WHO. *Weight Scale*, http://www.euro.who.int/en/health-topics/disease-prevention/nutrition/a-healthy-lifestyle/body-mass-index-bmi (2018).

[33] Dhaliwal SS, Welborn TA (2009). Central obesity and cigarette smoking are key determinants of cardiovascular disease deaths in Australia: a public health perspective. Prev Med.

[34] Lean ME (2000). Pathophysiology of obesity. Proc Nutr Soc.

[35] Schober P, Vetter TR (2019). Two-sample unpaired t tests in medical research. Anesth Analg.

[36] Schulte PJ, Mascha EJ (2018). Propensity score methods: theory and practice for anesthesia research. Anesth. Analg..

[37] Schober P, Vetter TR (2018). Repeated measures designs and analysis of longitudinal data: if at first you do not succeed-try, try again. Anesth. Analg..

[38] Schober, P. & Vetter, T. R. Confounding in observational research. *Anesth. Analg.* **in press** (2020). 10.1213/ANE.0000000000004627 32068590

[39] Vetter TR, Schober P (2018). Regression: the apple does not fall far from the tree. Anesth. Analg..

