# Supplementary material for: Rethinking the causes of pilonidal sinus disease: a matched cohort study
Source: Sci Rep. 2021 Mar 18;11:6210. doi: 10.1038/s41598-021-85830-1 (PMC7973489; doi:10.1038/s41598-021-85830-1)
Supplement: Supplementary file 1 — Supplementary Figure [file 41598_2021_85830_MOESM1_ESM.docx]

**Title: Rethinking the causes of pilonidal sinus disease: A matched cohort study – supplementary file**

**Short title: Rethinking PSD - supplementary file**

**Authors:** *Dietrich Doll, MD, PhD^1^; Imke Brengelmann, MD^1^; Patrick Schober, MD, PhD, MMedStat^2^; Andreas Ommer, MD^3^; Friederike Bosche, MD^1^; Apostolos E. Papalois, PhD^4^) ; Sven Petersen, MD, PhD^5^; Dirk Wilhelm, MD, PhD^6^; Johannes Jongen, MD^7^; Markus M. Luedi, MD^8^

**Affiliations:** ^1^Department of Procto-Surgery, St. Marienhospital Vechta, Academic Teaching Hospital of the MHH Hannover, Vechta, Germany (DD, IB, FB)

^2^Department of Anesthesiology, Amsterdam University Medical Centers, Vrije Universiteit Amsterdam, Amsterdam, Netherlands (PS)

^3^End- und Dickdarm-Zentrum Essen, Germany (AO)

^4^ELPEN Pharmaceutical Research & Experimental Center, Athens, Greece (AEP)

^5^Department of General, Visceral and Vascular Surgery, Asklepios Klinik Altona, Hamburg, Germany (SP)

^6^Department of Surgery, Klinikum Rechts der Isar, Munich, Germany (DW)

^7^Private Practice, Kiel, Germany (JJ)

^8^Department of Anaesthesiology and Pain Medicine, Inselspital, Bern University Hospital, University of Bern, Bern, Switzerland (MML)

**Correspondence to:** Prof. Dr. med. Dr. phil. Dietrich Doll, Department of Procto-Surgery, St Marienhospital Vechta, Academic Teaching Hospital of the Medical School Hannover, Vechta, Germany, Tel +49 4441 99 1240, Cell +49 1723 58 54 54, Fax +49 4441 99 1250, Dietrich.Doll@kk-om.de

**Supplemental Figure 1**

**Sweating test in pilonidal sinus patients:** Anatomical arrangement of the pilocarpine iontophoresis sweat test, with electrodes placed in the glabella sacralis.
